# Determinação da Idade Vascular em Homens Através do Escore de Cálcio Coronariano e seu Impacto na Reestratificação do Risco Cardiovascular

**DOI:** 10.36660/abc.20230253

**Published:** 2023-10-20

**Authors:** Ismael Polli, Neide Maria Bruscato, Protasio Lemos Da Luz, Douglas Dal Más Freitas, Angélica Oliveira de Almeida, Waldemar De Carli, Emilio Hideyuki Moriguchi

**Affiliations:** 1 Universidade Federal do Rio Grande do Sul Porto Alegre RS Brasil Universidade Federal do Rio Grande do Sul , Porto Alegre , RS – Brasil; 2 Hospital Comunitário São Peregrino Lazziozi AVAES Veranópolis RS Brasil Hospital Comunitário São Peregrino Lazziozi , Associação Veranense de Assistência em Saúde ( AVAES ), Veranópolis , RS – Brasil; 3 Instituto Moriguchi Veranópolis RS Brasil Instituto Moriguchi , Veranópolis , RS – Brasil; 4 Hospital das Clínicas Faculdade de Medicina Universidade de São Paulo São Paulo SP Brasil Instituto do Coração do Hospital das Clínicas da Faculdade de Medicina da Universidade de São Paulo , São Paulo , SP – Brasil

**Keywords:** Aterosclerose, Calcificação Vascular, Doenças Cardiovasculares

## Abstract

**Fundamento:**

Identificar os indivíduos assintomáticos sob risco de desenvolver doenças cardiovasculares é um dos principais objetivos da cardiologia preventiva. O escore de cálcio coronariano (ECC) permite estimar a idade vascular, que se mostrou mais fidedigna que a idade cronológica na determinação do risco cardiovascular.

**Objetivos:**

Reclassificar o risco cardiovascular com base na idade arterial e avaliar a progressão do escore de cálcio durante o seguimento.

**Métodos:**

150 homens assintomáticos foram submetidos a avaliação clínica e do ECC em 2 avaliações com intervalo de 7,6 anos. Classificamos os pacientes pelos escores de risco tradicionais e pela idade arterial. Avaliamos quais variáveis se associaram a maior progressão do ECC durante o período. O nível de significância estatística considerado foi de 5% (p < 0,05).

**Resultados:**

A utilização da idade arterial na estratificação do risco cardiovascular em comparação ao escore de risco de Framingham (ERF) reclassificou 29% dos indivíduos para uma categoria de risco superior e 37% para uma categoria inferior. Em relação ao escore da AHA e ACC (ASCVD), 31% passaram para um risco maior e 36% para um risco menor. A classificação inicial pela idade arterial teve relação direta com a progressão do ECC ao longo do seguimento (p < 0,001), fato que não foi observado para o ERF (p = 0,862) e ASCVD (p = 0,153). As variáveis individuais que mais se associaram à progressão do ECC foram a pressão arterial sistólica e o HDL baixo.

**Conclusão:**

A estratificação de risco cardiovascular utilizando a idade arterial apresentou melhor associação que o ERF e ASCVD na identificação de indivíduos com maior risco de progressão da aterosclerose.

## Figura Central

**Figure:**
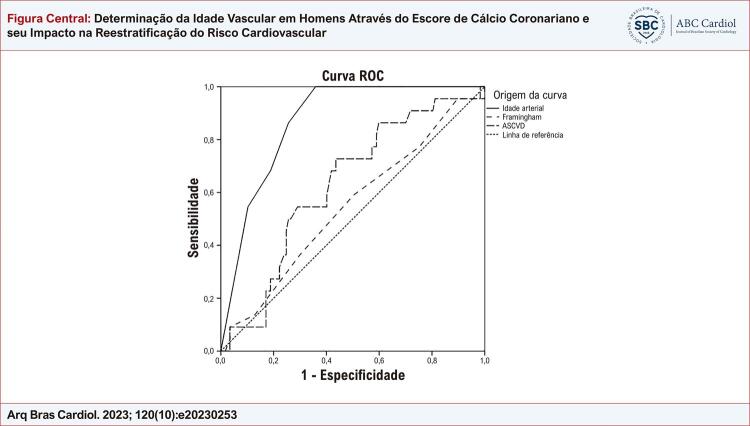
: Determinação da Idade Vascular em Homens Através do Escore de Cálcio Coronariano e seu Impacto na Reestratificação do Risco Cardiovascular

Curva ROC comparando a classificação pela idade arterial com ASCVD e ERF.

## Introdução

A aterosclerose inicia em uma fase subclínica e avança lentamente ao longo de anos antes do desenvolvimento de sintomas ou eventos clínicos cardiovasculares. ^1^

A capacidade de identificar dentre os indivíduos assintomáticos o subgrupo que apresenta maior risco de desenvolver eventos cardiovasculares é uma estratégia fundamental na prevenção de eventos cardiovasculares. ^2^

Os escores de risco clínico como o escore de risco de Framingham (ERF) são amplamente utilizados para a estratificação dos indivíduos com maior risco cardiovascular. ^3^ Entretanto, estima-se que o risco em muitos indivíduos possa ser subestimado, tendo em vista que a aterosclerose é uma doença heterogênea que se desenvolve de maneira não uniforme entre os indivíduos. Além disso, uma grande porcentagem é classificada como risco intermediário, onde as condutas terapêuticas são controversas. ^4 - 7^

A tomografia computadorizada permite a mensuração do escore de cálcio coronariano (ECC) e, a partir desse dado é possível estimar a idade arterial, ou seja, baseado no dano já presente nos vasos analisados, é possível estimar a idade dos mesmos. A utilização da idade arterial no ERF mostrou-se mais preditiva de eventos futuros do que o uso da idade cronológica. ^8 - 11^

O estudo MESA (The *Multi* -Ethnic Study of Atherosclerosis) avaliou o impacto da determinação do ECC na predição dos eventos coronarianos em 6.722 homens e mulheres de diversas etnias. Em comparação com aqueles indivíduos sem calcificação coronária, o risco de morte ou infarto agudo do miocárdio, aumentou em 7,7 vezes para os indivíduos com ECC entre 101 e 300 e 9,7 vezes para ECC > 300. ^12^

O uso da idade arterial ao invés da idade cronológica reclassificou 28% dos participantes do estudo MESA para categorias de risco diferentes do original. Durante a análise de desfechos, o escore baseado na idade arterial tem um desempenho significativamente melhor para determinação do risco (área sob a curva ROC 0,75 para o ERF com base na idade observada versus 0,79 usando a idade arterial). ^11^

Buscamos no presente estudo reclassificar o risco cardiovascular com base na idade arterial e avaliar a progressão do escore de cálcio durante o seguimento.

## Métodos

### População estudada

O Projeto Estudos dos Índices de Envelhecimento e Prevalência de Aterosclerose em Bebedores de Vinho Habituais versus Abstêmios é um estudo transversal de base populacional, tendo como objetivo principal avaliar os índices de envelhecimento arterial e prevalência de aterosclerose em bebedores de vinho habituais versus abstêmios. O protocolo do estudo foi desenvolvido pelo Instituto do Coração de São Paulo (INCOR–SP), Brasil. O estudo foi desenvolvido em São Paulo, com uma amostra complementar em Veranópolis, Rio Grande do Sul (RS), Brasil. Parte do estudo desenvolvido em São Paulo teve a descrição detalhada do desenho do estudo e métodos publicados anteriormente. ^13^

A amostra complementar desenvolvida em Veranópolis foi com uma amostra diferenciada em relação ao consumo do vinho tinto, sendo este um consumo habitual e não de confrarias, como observado na amostra de São Paulo. A descrição detalhada do estudo e métodos foi publicada anteriormente. ^14^

Para o presente estudo, foram utilizados exclusivamente dados referentes à amostra de Veranópolis, sendo a primeira avaliação realizada em 2011 e a segunda em 2019, com intervalo médio de 7,6 anos. Na primeira etapa foram recrutados 150 voluntários brancos do sexo masculino entre 50 e 70 anos. A segunda etapa incluiu 139 participantes da amostra inicial. Em ambas as etapas os indivíduos foram submetidos a avaliação clínica, exames laboratoriais e tomografia para avaliar o ECC.

Foram incluídos apenas homens no estudo devido ao estudo original abordar consumo regular de vinho e tradicionalmente esse hábito é mais comum em homens na área geográfica estudada.

O projeto de pesquisa foi aprovado pelo Comitê de Ética em Pesquisa do Hospital de Clínicas de Porto Alegre RS, em 07 de outubro de 2020 sob número 4.325.574.

### Fatores de risco cardiovascular e questionário

Todos os dados foram coletados por um questionário estruturado no estudo-base e repetido na segunda etapa no Hospital Comunitário São Peregrino Lazziozi em Veranópolis, RS, Brasil. Todos os participantes do estudo responderam sobre dados de identificação, aspectos demográficos, características clínicas e fatores de risco cardiovascular. As coletas de sangue para os exames de perfil lipídico e glicemia foram realizadas com jejum de, no mínimo, 12 horas em laboratório, após a assinatura do termo de consentimento livre e esclarecido.

A escolaridade foi classificada em 3 categorias: (≤ 8 anos, 9 a 12 anos e > 12 anos). Em relação à renda, os participantes foram classificados em: < 5 salários mínimos, de 5 a 10 salários mínimos e > 10 salários mínimos em moeda brasileira.

A atividade física foi avaliada em relação ao tempo e periodicidade semanal em duas categorias: < 150 minutos/semana e ≥ 150 minutos/semana, de acordo com as diretrizes da American College of Sports Medicine e American Heart Association (ACSM/AHA). ^15^

Para o índice de massa corporal (IMC) foi utilizado o índice de Quetelet (IMC = peso/altura ^2^ ). ^16^

A pressão arterial foi medida no braço direito, com o indivíduo sentado, através de esfigmomanômetro tipo aneroide, previamente aferido por instituição credenciada pelo Inmetro Brasil. Foi utilizado o valor médio entre 2 medidas com 5 minutos de intervalo entre elas, após um repouso de 10 minutos do participante. A hipertensão foi definida como níveis ≥ 140 mmHg para pressão arterial sistólica e/ou ≥ 90 mmHg para pressão arterial diastólica, e/ou estar em uso de medicação anti-hipertensiva. ^17^

O diabetes mellitus foi definido como glicemia de jejum ≥ 126 mg/dL ou em uso de medicamentos hipoglicemiantes. ^18^

A história familiar de doença arterial coronariana prematura foi considerada positiva quando algum membro de sua família, pai ou mãe ou ambos, com idade inferior aos 55 anos de idade se do sexo masculino ou inferior aos 65 anos de idade se do sexo feminino, tinha sofrido infarto do miocárdio fatal ou não, e/ou angioplastia coronária ou cirurgia de revascularização miocárdica. ^19^

### Avaliação da calcificação coronária

Foi realizada através de um equipamento de tomografia computadorizada de tórax. Na primeira fase foi utilizado Tomógrafo Somaton Sensation 64 da Siemens com 64 detectores e na segunda fase o Tomógrafo Siemens Drive® com 256 detectores, no Hospital Moinhos de Vento em Porto Alegre, RS, Brasil. Na aquisição do escore de cálcio, a espessura de cortes considerada foi de 3 mm. A calcificação das artérias coronárias foi avaliada pelo escore de Agatston. ^20^

A pontuação do ECC no estudo foi calculada pelo método Agatston e classificada como 0, 1-101, 101-400, 401-1000 e > 1000. ^21^ Para facilitar a interpretação e prevalência dos dados, as categorias de pontuação do ECC foram classificadas nas seguintes categorias: sem evidência (ECC = 0), ECC discreto (1-100), ECC moderado (101-400), ECC alto (401-1000) e muito alto (> 1000).

### Idade arterial

A idade arterial é uma maneira mais prática de transformar a pontuação do escore de cálcio de unidades Agatston para unidades etárias de maneira que ficasse mais prática para uso e entendimento de médicos e pacientes. Ou seja, através do banco de dados obtido no estudo MESA, os pesquisadores observaram qual seria o nível mais prevalente de ECC para cada faixa etária e, dessa forma, estimou-se a idade da artéria daquele indivíduo. Para estimar o risco cardiovascular, recomendam utilizar a idade arterial ao invés da idade cronológica no ERF. ^11^

### Escores de risco

As variáveis obtidas nas duas etapas do estudo foram utilizadas para estimar o risco cardiovascular em 10 anos pelo ERF e pelo ASCVD. ^22 , 23^ Além dessas variáveis, o ECC foi utilizado para o cálculo da idade arterial através da calculadora online do estudo MESA (http://www.mesa-nhlbi.org/Calcium/ArterialAge). ^24^

Os indivíduos foram classificados como baixo risco (menos de 10% de risco absoluto de eventos cardiovasculares maiores em 10 anos), risco intermediário (entre 10% e 20%) e alto risco (mais que 20% em 10 anos). Para o escore de ASCVD, optamos por agrupar os indivíduos de baixo risco com os classificados com o borderline de maneira a simplificar a análise. Desse modo esses indivíduos foram classificados como baixo risco (risco estimado até 7,4%), risco intermediário (7,5% a 19,9%) e alto risco (maior que 20%).

Analisamos a proporção de indivíduos que migrou de uma categoria de risco para outra quando utilizada a idade arterial, bem como avaliamos individualmente cada variável a fim de identificar as que tiveram maior associação com a progressão do escore de cálcio no período de acompanhamento.

### Análise estatística

As variáveis quantitativas foram descritas por média e desvio padrão ou mediana e amplitude interquartílica, conforme normalidade dos dados. As variáveis categóricas foram descritas por frequências absolutas e relativas.

Para comparar medianas da variação do escore de cálcio, os testes de Mann-Whitney ou Kruskal-Wallis complementado por Dunn foram aplicados. A normalidade foi verificada pelo teste de Kolmogorov-Smirnov.

As associações com as variáveis quantitativas e ordinais foram avaliadas pelo coeficiente de correlação de Spearman. A concordância entre as 3 classificações de risco foi avaliada pelo coeficiente kappa.

A idade arterial, o ERF e ASCVD foram avaliados numericamente através da curva ROC (receiver operating characteristic) em relação ao escore de cálcio maior do que 400 na segunda avaliação.

O nível de significância estatística considerado foi de 5% (p < 0,05). O software utilizado para a análise dos dados foi o SPSS (Statistical Package for the Social Sciences), versão 18.0.

## Resultados

A amostra foi constituída de 150 homens com média de idade de 58,2 anos ( Tabela 1 ).

**Tabela 1:** – Características dos participantes do estudo

Variáveis	Primeira etapa (n=150)	Segunda etapa (n=139)
Idade (anos)	58,2 ± 5,3	65,7 ± 5,2
Anos de estudo – n (%)		
≤ 8	86 (57,3)	79 (56,83)
9-12	39 (26,0)	37 (26,6)
> 12	25 (16,7)	23 (16,5)
Renda – n (%)		
< 5 salários	103 (68,7)	93 (66,9)
5 a 10 salários	37 (24,7)	40 (28,8)
> 10 salários	10 (6,7)	6 (4,3)
IMC (kg/m ^2^ )	26,8 ± 2,5	27,3 ± 3,4
Circunferência da cintura (cm)	96,3 ± 7,9	97,7 ± 9,0
PAS (mmHg)	139,8 ± 12,2	137,36 ± 17,7
PAD (mmHg)	83,6 ± 7,8	78,1 ± 9,7
FC (bpm)	65,5 ± 10,7	66,4 ± 9,6
Tabagista – n (%)	18 (12,0)	5 (3,6)
História familiar de DAC – n (%)	23 (15,3)	40 (28,8)
Atividade física semanal – n (%)		
< 150 min	25 (16,7)	38 (27,3)
> 150 min	125 (83,3)	101 (72,7)
Exame físico alterado – n (%)	14 (9,3)	17 (12,3)
Glicose (mg/dL)	106,0 ± 17,4	100,3 ± 19,5
Colesterol total (mg/dL)	226,6 ± 38,0	195,7 ± 40,8
Colesterol LDL (mg/dL)	145,2 ± 32,8	120,6 ± 36,9
Colesterol HDL (mg/dL)	49,2 ± 13,2	50,2 ± 11,7
Triglicerídeos (mg/dL)	163,9 ± 141,2	124 ± 72,4
Escore de cálcio coronariano	94,4 ± 218,5 (0-83)	228,6 ± 468,5 (0-213)
Sem evidência	61 (40,7)	40 (28,7)
Discreto (1-100)	57 (38,0)	52 (37,4)
Moderado (101-400)	21 (14,0)	25 (18,0)
Alto (401-1000)	10 (6,6)	14 (10,1)
Muito alto (> 1000)	1 (0,7)	8 (5,7)

* Dados quantitativos são descritos por média ± desvio padrão ou mediana (percentis 25-75) e categóricos por n (%). DAC: doença arterial coronariana; FC: frequência cardíaca; IMC: índice de massa corporal; PAD: pressão arterial diastólica; PAS: pressão arterial sistólica.

Ao longo do estudo foram perdidos 11 acompanhamentos, sendo 6 óbitos (nenhum caso de morte de causa cardiovascular) e 5 que se negaram a participar, restando para a segunda etapa 139 indivíduos.

O cálculo da idade arterial mostrou uma diferença de 1,7 anos a menos em relação à idade cronológica, sendo estimada a idade arterial média de 56,5 anos versus 58,2 anos da idade cronológica. Na segunda etapa essa diferença foi ampliada para 3 anos.

A reestratificação do risco cardiovascular utilizando-se a idade arterial apresentou diferença estatisticamente significativa em relação à estratificação de risco cardiovascular pelos ERF e ASCVD. Observamos muitos indivíduos que foram reclassificados, tanto para maior quanto para menor risco.

Em relação ao ERF, 29% dos indivíduos foram reclassificados para uma categoria de risco superior ao utilizar a idade arterial, enquanto 34% permaneceram na mesma categoria e 37% passaram para uma categoria inferior de risco. Para o ASCVD os achados foram semelhantes, sendo que 33% permaneceram na mesma categoria, enquanto 31% passaram para um risco maior e 36% para um risco menor.

**Figura 1:**
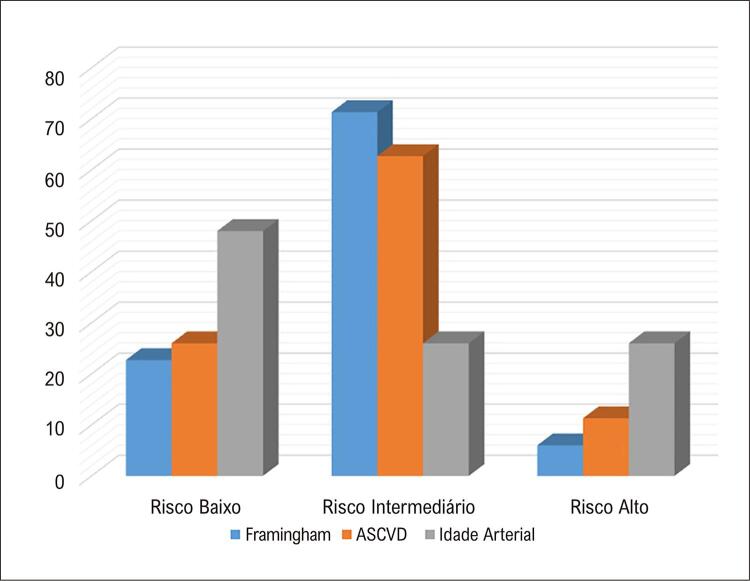
– Reclassificação das categorias de risco com os diferentes escores na primeira avaliação.

A maior diferença foi observada nos indivíduos de risco intermediário por Framingham em que dos 107 indivíduos inicialmente agrupados nessa categoria apenas 27 (25,2%) permaneceram em uma escala de risco intermediário, enquanto 52 (48,5%) foram reclassificados para baixo risco e 28 (26,2%) para alto risco ( Figura 1 ).

Quando avaliadas individualmente as categorias de risco pela classificação do ASCVD, na fase inicial havia 39 indivíduos classificados como baixo risco, dos quais 22 (56%) mudaram para uma categoria mais alta. Para os de alto risco pelo ASCVD, dos 17 inicialmente classificados, apenas 5 permaneceram (29%) como alto risco após a inclusão do escore de cálcio.

**Figura 2:**
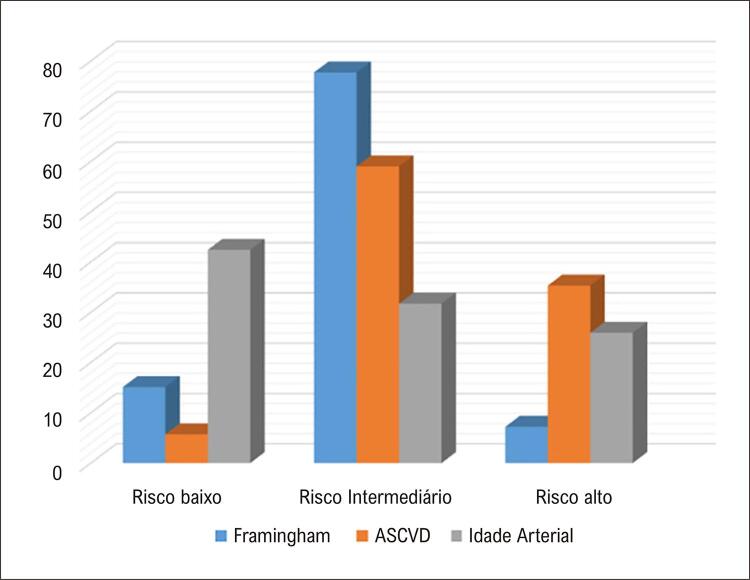
– Reclassificação das categorias de risco com os diferentes escores na segunda avaliação.

Na segunda etapa do estudo, com tempo médio de 7,6 anos após a primeira avaliação, ocorreu um fenômeno semelhante. Naqueles indivíduos de risco intermediário por Framingham a porcentagem que permaneceu com a mesma classificação foi de 30,5%, enquanto 39,8% foram reclassificados para baixo risco e 29,6% para alto risco ( Figura 2 ).

Como exemplo, citamos um caso de um indivíduo de 52 anos de idade com colesterol total de 270 mg/dL, HDL-colesterol de 43 mg/dL, pressão arterial sistólica de 129 mmHg e diastólica de 87 mmHg, sem histórico de tabagismo e não diabético. Ao aplicarmos o ERF o risco estimado foi de 8% (baixo risco), ASCVD 7,3 (borderline). Entretanto, o ECC foi de 195 o que elevou seu risco pela idade arterial para 30, portanto passou a ser considerado como alto risco de doenças cardiovasculares. E, de fato, no acompanhamento, seu ECC passou a 631, um incremento de 323% e somente aí passa a ser considerado como alto risco pelos critérios clínicos.

Avaliou-se também a concordância entre os três escores (ERF, ASCVD e idade arterial) para classificar risco, e foi observado que o coeficiente kappa entre idade arterial e Framingham foi de 0,044 (p = 0,330), o kappa entre idade arterial e ASCVD foi de 0,073 (p = 0,080) e entre Framingham e ASCVD foi de 0,107 (p < 0,001), demonstrando concordância significativa apenas entre as classificações de risco de Framingham e ASCVD, mas com baixa concordância, apesar de significativa. Ou seja, ERF e ASCVD classificaram de maneira semelhante os grupos de risco, entretanto ambas diferiram da classificação pela idade arterial.

Houve aumento significativo do escore de cálcio no período entre as 2 avaliações (primeira avaliação: mediana = 12,5; percentis 25-75: 0-81; segunda avaliação: mediana = 33; percentis 25-75: 0-213; p < 0,001). A mediana da variação do escore de cálcio entre as 2 avaliações foi de 14 pontos (percentis 25-75: 0-116).

Ao fazer a análise progressiva do delta de variação do escore de cálcio, ou seja, ao avaliar o quanto o escore de cálcio varia ao longo do período de seguimento, observamos que os indivíduos classificados inicialmente como baixo risco pela idade arterial tiveram uma progressão muito pequena no escore de cálcio, e essa progressão aumentou proporcionalmente quanto maior o risco estimado pela classificação inicial (p < 0,001). Essa relação não foi significativa para o ERF (p = 0,862) nem para ASCVD (p = 0,153). As diferenças entre as classificações de risco com o aumento do escore de cálcio estão apresentadas na Tabela 2 .

**Tabela 2:** – Diferenças entre as classificações de risco com a variação do escore de cálcio

Variáveis	Amostra total n (%)	∆ Escore de cálcio Mediana (P25-P75)	p
**Classificação do risco estimado pela idade arterial**			< 0,001
< 10 - risco baixo	72 (48,0)	0 (0-11,8) ^a^	
10 a 20 - risco moderado	39 (26,0)	38,5 (6-246) ^b^	
> 20 - risco elevado	39 (26,0)	148 (58,5-466) ^c^	
**Classificação Framingham**			0,862
< 10% - risco baixo	34 (22,7)	12 (0-76,5)	
10% a 20% - risco intermediário	107 (71,3)	18,5 (0-126)	
> 20% - risco elevado	9 (6,0)	47 (1-542)	
**ASCVD**			0,153
< 5% - risco baixo	39 (26,0)	8,5 (0-35,0)	
5% a 19,9% - risco intermediário	94 (62,7)	25 (0-173)	
≥ 20% - risco elevado	17 (11,3)	30,6 (0,5-60,5)	

^a,b,c^ Letras iguais não diferem pelo teste de Dunn a 5% de significância. Os números apresentados são relativos à pontuação de acordo com o método de Agatston.

**Figura 3:**
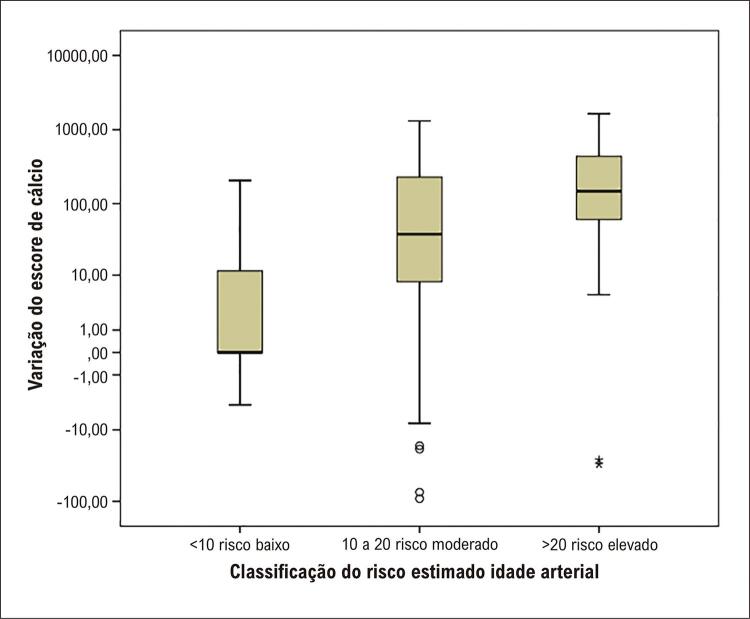
– Variação do escore de cálcio conforme classificação do risco estimado pela idade arterial. A linha dentro da caixa representa a mediana. Os limites inferior e superior da caixa representam os percentis 25 e 75, respectivamente. As barras de erro inferior e superior representam os valores mínimo e máximo estimados para o grupo. Os círculos e asteriscos demarcam os valores extremos da amostra.

A Figura 3 ilustra as diferenças encontradas na variação do escore de cálcio conforme aumento do risco cardiovascular pela idade arterial.

A classificação numérica (% de risco cardiovascular) estimada na primeira avaliação pelos diferentes métodos (classificação de risco pela idade arterial, por ERF e ASCVD) foi comparada através da curva ROC para o desfecho ECC > 400 na segunda avaliação, valor que é considerado como alto grau de calcificação coronariana. Ou seja, objetivamos avaliar a sensibilidade e especificidade dos métodos como forma de detectar os indivíduos que viriam a desenvolver maior grau de calcificação coronariana no período de acompanhamento (7,6 anos).

Observamos que a classificação pela idade arterial foi significativamente superior que os demais métodos, com área sob a curva de 0,870, enquanto ASCVD apresentou área sob a curva de 0,629 e o ERF praticamente ficou na linha de base, com 0,544 ( Figura Central ).

A avaliação individualizada das variáveis analisadas no estudo mostrou que a grande maioria não teve relação significativamente estatística com a variação do escore de cálcio. Não foi observada essa relação para: idade, presença de hipertensão arterial sistêmica, uso de estatinas, diabetes mellitus, LDL elevado, hipertrigliceridemia, tabagismo ou ex-tabagismo, atividade física, consumo de álcool, nível de escolaridade, estado civil, renda, IMC e circunferência da cintura. Dessa maneira, diminuímos a possibilidade de vieses pelos fatores confundidores.

Houve relação positiva estatisticamente significativa entre os níveis pressóricos sistólicos com a variação do escore de cálcio (p = 0,041), sendo que quanto maiores os níveis da pressão arterial sistólica na avaliação basal, maior o aumento no escore de cálcio ( Tabela 3 ).

**Tabela 3:** – Associações das variáveis com a variação do escore de cálcio

Variável quantitativa	∆ Escore de cálcio Coeficiente de correlação de Spearman	p
PAS	0,174	0,041
**Variável qualitativa**	**Mediana (P25-P75)**	**p**
HDL baixo		0,041
Não	13 (0-114)	
Sim	457,5 (368-547)	

PAS: pressão arterial sistólica.

Indivíduos com HDL baixo apresentaram significativamente maior aumento no escore de cálcio, conforme apresenta a Tabela 3 . A média de aumento do escore de cálcio ao longo do estudo foi de 457, enquanto nos demais indivíduos com HDL normal, a média de aumento no escore de cálcio foi de 13.

## Discussão

O ECC é indicador de aterosclerose subclínica e o seu valor elevado significa maior grau de aterosclerose. ^9 , 11 , 12^

O impacto dos fatores de risco tradicionais não é uniforme entre os indivíduos, por isso escores tradicionais nem sempre apresentam sensibilidade e especificidade suficientes para a correta estratificação de risco. ^4 - 6^

Os estudos mostram que incorporar a idade arterial aos escores de risco tradicionais eleva a área sob a curva, fato que melhora a sensibilidade e a especificidade desse escore na determinação do risco cardiovascular. ^11 , 25^

Nosso estudo mostrou que uma parcela significativa dos indivíduos é reestratificada para categorias de risco diferentes ao ser avaliada através da idade arterial. A porcentagem que muda de categoria de risco foi maior que a observada no PAAC study ^26^ e no estudo Heinz Nixdorf Recall Study, ^27^ avaliação derivada do estudo MESA. A categoria que mais sofre alterações é a classificada como intermediária pelos escores de risco tradicionais, fato semelhante aos estudos citados anteriormente, onde entre 1 a cada 4 e 1 a cada 6 indivíduos passam para uma categoria de risco superior. Em nosso estudo observamos um número maior de indivíduos que passa para uma categoria de risco inferior em comparação aos estudos citados.

Tais achados reforçam a indicação já existente nas diretrizes para que seja realizada a determinação do ECC especialmente naqueles indivíduos com risco cardiovascular intermediário pelos escores de risco tradicionais. ^22 , 28 , 29^

Além disso, o dado mais objetivo foi que a classificação do risco cardiovascular pela idade arterial mostrou relação direta com o aumento da calcificação coronariana determinada através do ECC ao longo do acompanhamento. Indivíduos classificados inicialmente como baixo risco pela idade arterial tiveram uma progressão muito pequena no ECC, e essa progressão aumenta proporcionalmente quanto maior o risco estimado pela classificação inicial. Essa relação já havia sido descrita na literatura para desfechos cardiovasculares maiores, entretanto não há descrição do aumento proporcional do ECC com base na classificação inicial pela idade arterial. Esse é o dado mais relevante do nosso estudo e pode indicar a realização de ECC mesmo em indivíduos de baixo risco pelos escores tradicionais, tendo em vista que mesmo dentre esses indivíduos, alguns possuíam idade arterial mais elevada e tiveram progressão mais significativa do escore de cálcio ao longo do acompanhamento.

Entre todos os fatores de risco estudados, a pressão arterial sistólica e o HDL baixo foram os que se associaram de maneira significativa com a maior progressão do ECC ao longo do estudo.

O HDL colesterol já se mostrou um importante fator de risco cardiovascular quando em níveis baixos, independente dos demais níveis de colesterol. ^30 , 31^ Em nosso estudo foi o fator isolado com maior impacto na progressão da calcificação coronariana.

Embora ocorra uma significativa reclassificação do risco cardiovascular, não foi possível avaliar a potencial redução de desfechos cardiovasculares nessa população estudada. Esse fato nos faz questionar a aplicabilidade do método para a população em geral, tendo em vista que os custos e os potenciais riscos associados ao exame não podem ser desprezados.

As principais limitações do estudo são o número relativamente baixo de participantes, ausência de mulheres na amostra e a incapacidade de mostrar desfechos cardiovasculares maiores (infarto agudo do miocárdio, morte de origem cardiovascular ou acidente vascular cerebral). Além disso, os participantes foram voluntários e selecionados por conveniência.

Um dos potenciais vieses a serem considerados é o uso de estatinas e seu possível efeito sobre o cálcio coronariano. Está estabelecido que o uso de estatinas pode aumentar o ECC, e desse modo a progressão do ECC nem sempre é algo indesejável, uma vez que pode apenas traduzir “estabilização” das placas coronarianas existentes. Possuíamos a informação dos pacientes que usavam estatinas nas duas etapas do estudo, embora não tenha sido obtida informação sobre o tempo e a continuidade de uso.

Além disso, os equipamentos utilizados nas duas etapas do estudo foram distintos, o que poderia inferir um potencial viés de aferição.

Uma limitação importante a ser considerada é a ausência de mulheres no estudo. Sabemos da importância de incluir ambos os gêneros para uma análise mais fidedigna e para que os dados possam ser generalizados. Pesquisas futuras devem ser realizadas incluindo mulheres para que possamos ter uma compreensão mais abrangente dos fatores que influenciam a idade vascular e as classificações de risco cardiovascular.

Embora os potenciais vieses, consideramos que o fator progressão do ECC foi bastante significativo e teve relação direta com a classificação inicial embasada na idade arterial. Além disso, pressão arterial sistólica elevada e níveis baixos de HDL-colesterol se associaram a maior progressão do ECC.

## Conclusão

A estratificação de risco cardiovascular utilizando a idade arterial teve maior capacidade de identificar os indivíduos com risco elevado de progressão da aterosclerose medida pelo ECC do que os escores de risco tradicionais (ERF e ASCVD). As variáveis individuais que mais se associaram à progressão do ECC foram a pressão arterial sistólica e o HDL baixo.
