# Supplementary material for: Determinação da Idade Vascular em Homens Através do Escore de Cálcio Coronariano e seu Impacto na Reestratificação do Risco Cardiovascular
Source: Arq Bras Cardiol. 2023 Oct 20;120(10):e20230253. [Article in Portuguese] doi: 10.36660/abc.20230253 (PMC10593387; doi:10.36660/abc.20230253)
Supplement: Translation

# Translation: Determination of Vascular Age in Men Using the Coronary Calcium Score and its Impact on Restratification of Cardiovascular Risk

**Authors:** Ismael Polli, Neide Maria Bruscato, Protasio Lemos Da Luz, Douglas Dal Más Freitas, Angélica Oliveira de Almeida, Waldemar De Carli, Emilio Hideyuki Moriguchi

**Affiliations:** 1 Brasil Universidade Federal do Rio Grande do Sul, Porto Alegre, RS – Brasil; 2 Brasil Hospital Comunitário São Peregrino Lazziozi, Associação Veranense de Assistência em Saúde (AVAES), Veranópolis, RS – Brasil; 3 Brasil Instituto Moriguchi, Veranópolis, RS – Brasil; 4 Brasil Instituto do Coração do Hospital das Clínicas da Faculdade de Medicina da Universidade de São Paulo, São Paulo, SP – Brasil

**Keywords:** Atherosclerosis, Vascular Calcification, Cardiovascular Diseases

## Abstract

**Background:**

Identifying asymptomatic individuals at risk of developing cardiovascular disease is one of the main goals of preventive cardiology. The coronary calcium score (CCS) makes it possible to estimate vascular age, which has shown to be more reliable than chronological age for determining cardiovascular risk.

**Objectives:**

To reclassify cardiovascular risk based on arterial age and evaluate CCS progression during follow-up.

**Methods:**

We included 150 asymptomatic men who underwent clinical and CCS evaluation in 2 evaluations with an interval of 7.6 years. We classified patients by traditional risk scores and arterial age. We evaluated which variables were associated with greater CCS progression during the period, considering a statistical significance level of 5% (p < 0.05).

**Results:**

The use of arterial age in the stratification of cardiovascular risk in comparison with the Framingham risk score (FRS) reclassified 29% of individuals to a higher risk category and 37% to a lower risk category. Regarding the American Heart Association and American College of Cardiology score (ASCVD), 31% were reclassified as higher risk and 36% as lower risk. The initial classification by arterial age was directly related to the progression of CCS throughout follow-up (p < 0.001). This was not observed for the FRS (p = 0.862) or ASCVD (p = 0.153). The individual variables most associated with CCS progression were high systolic blood pressure and low HDL.

**Conclusion:**

Cardiovascular risk stratification using arterial age showed a better association than the FRS and ASCVD in identifying individuals with higher risk of atherosclerosis progression.

## Introduction

Atherosclerosis begins in a subclinical phase and slowly progresses over the years before the development of symptoms or clinical cardiovascular events. ^1^

The ability to identify among asymptomatic individuals the subgroup that has a greater risk of developing cardiovascular events is a fundamental strategy in the prevention of cardiovascular events. ^2^

Clinical risk scores, such as the Framingham risk score (FRS), are widely used for stratifying individuals with greater cardiovascular risk. ^3^ However, it has been suggested that the risk in many individuals may be underestimated, considering that atherosclerosis is a heterogeneous disease that develops unevenly among individuals. In addition, a large percentage is classified as intermediate risk, where therapeutic approaches are controversial. ^4 - 7^

Computed tomography makes it possible to measure the coronary calcium score (CCS), and, based on this data, it is possible to estimate arterial age, that is, based on the damage already present in the analyzed vessels, it is possible to estimate their age. Using arterial age in the Framingham risk score has shown to be more predictive of future events than chronological age. ^8 - 11^

The *Multi* -Ethnic Study of Atherosclerosis (MESA) evaluated the impact of determining CCS on the prediction of coronary events in 6,722 men and women of diverse ethnicities. In comparison with individuals without coronary calcification, the risk of death or acute myocardial infarction increased by 7.7 times for individuals with CCS between 101 and 300 and 9.7 times for CCS > 300. ^12^

Using arterial age rather than chronological age reclassified 28% of MESA participants into risk categories other than the original. During outcome analysis, the score based on arterial age performed significantly better for risk determination (area under the receiver operating characteristic [ROC] curve 0.75 for FRS based on observed age versus 0.79 using arterial age). ^11^

The objective of this study was to reclassify cardiovascular risk based on arterial age and to evaluate CCS progression during follow-up.

## Methods

### Study population

The Studies of Aging Indices and Prevalence of Atherosclerosis in Regular Wine Consumers versus Abstainers Project is a population-based cross-sectional study, whose main objective is to evaluate indices of arterial aging and the prevalence of atherosclerosis in habitual wine drinkers versus abstainers. The study protocol was developed by the São Paulo Heart Institute (INCOR–SP), Brazil. The study was developed in São Paulo, with a complementary sample in Veranópolis, Rio Grande do Sul, Brazil. Part of the study developed in São Paulo has had a detailed description of the study design and methods previously published. ^13^

The complementary sample developed in Veranópolis had a differentiated sample in relation to the consumption of red wine, being regular consumption and not only on social occasions, as observed in the sample from São Paulo. A detailed description of the study and methods has been previously published. ^14^

The present study exclusively used data referring to the sample from Veranópolis, with the first evaluation carried out in 2011 and the second in 2019, with an average interval of 7.6 years. During the first stage, 150 White male volunteers between 50 and 70 years of age were recruited. The second stage included 139 participants from the initial sample. In both stages, individuals underwent clinical evaluation, laboratory tests, and tomography to evaluate the CCS.

Only men were included in the study, because the original study addressed regular wine consumption, and this habit is traditionally more common among men in the geographical area studied.

The research project was approved by the Research Ethics Committee of the Hospital de Clínicas de Porto Alegre, Rio Grande do Sul, on 7 October 2020 under number 4.325.574.

### Cardiovascular risk factors and questionnaire

All data were collected using a structured questionnaire in the baseline study and repeated during the second stage at the São Peregrino Lazziozi Community Hospital in Veranópolis, Rio Grande do Sul, Brazil. All study participants answered questions regarding identification, demographic aspects, clinical characteristics, and cardiovascular risk factors. Blood collections for the lipid profile and blood glucose tests were performed after fasting for at least 12 hours in the laboratory, after signing the free and informed consent form.

Level of schooling was classified into the following 3 categories: (≤ 8 years, 9 to 12 years, and > 12 years). In relation to income, participants were classified as follows: < 5 times minimum wage, from 5 to 10 times minimum wage, and > 10 times minimum wage in Brazilian currency.

Physical activity was evaluated in relation to time and weekly frequency using the following 2 categories: < 150 minutes/week and ≥ 150 minutes/week, in accordance with the guidelines of the American College of Sports Medicine and American Heart Association (ACSM/AHA). ^15^

For body mass index (BMI), the Quetelet index was used (BMI = weight/height ^2^ ). ^16^

Blood pressure was measured in the right arm, with the individual sitting, using an aneroid sphygmomanometer, previously calibrated by an institution accredited by Inmetro, Brazil. We used the average value between 2 measurements, with a 5-minute interval between them, after the participant had rested for 10 minutes. Hypertension was defined as levels ≥ 140 mmHg for systolic blood pressure, and/or ≥ 90 mmHg for diastolic blood pressure, and/or being on antihypertensive medication. ^17^

Diabetes mellitus was defined as fasting blood glucose ≥ 126 mg/dL or use of hypoglycemic drugs. ^18^

Family history of premature coronary artery disease was considered positive when a family member (father, mother, or both), under the age of 55 years if male or under the age of 65 years if female, had suffered a fatal or non-fatal myocardial infarction and/or undergone coronary angioplasty or coronary artery bypass graft surgery. ^19^

### Evaluation of coronary calcification

Coronary calcification was evaluated using equipment for chest computed tomography. During the first phase, a Siemens Somaton Sensation 64 tomography scanner with 64 detectors was used, and, during the second phase, a Siemens Drive® tomography scanner with 256 detectors was used, at Hospital Moinhos de Vento in Porto Alegre, Rio Grande do Sul, Brazil. When acquiring the CCS, a slice thickness of 3 mm was considered. Calcification of the coronary arteries was assessed using the Agatston score. ^20^

The CCS in the study was calculated using the Agatston method and classified as 0, 1 to 101, 101 to 400, 401 to 1000, and > 1000. ^21^ To facilitate the interpretation and prevalence of the data, the CCS categories were classified as follows: no evidence (CCS = 0), mild CCS (1 to 100), moderate CCS (101 to 400), high CCS (401 to 1000), and very high (> 1000).

### Arterial age

Arterial age is a more practical means of transforming the CCS from Agatston units to age units in a manner that is more practical for physicians and patients to use and understand. In other words, through the database obtained by the MESA study, the researchers observed which would be the most prevalent level of CCS for each age group and, accordingly, estimated the age of those individuals’ arteries. To estimate cardiovascular risk, it has been recommended to use arterial age instead of chronological age in the FRS. ^11^

### Risk scores

The variables obtained in the 2 stages of the study were used to estimate the 10-year cardiovascular risk using the FRS and the ASCVD. ^22 , 23^ In addition to these variables, the CCS was used to calculate the arterial age using the online calculator of the MESA study (http://www.mesa-nhlbi.org/Calcium/ArterialAge). ^24^

Subjects were classified as low risk (less than 10% absolute risk of major cardiovascular events in 10 years), intermediate risk (between 10% and 20%), and high risk (greater than 20% in 10 years). For the ASCVD score, we opted to group low-risk individuals with those classified as borderline in order to simplify the analysis. Thus, individuals were classified as low risk (estimated risk up to 7.4%), intermediate risk (7.5% to 19.9%), and high risk (greater than 20%).

We analyzed the proportion of individuals who moved from one risk category to another when arterial age was used, as well as individually, evaluating each variable in order to identify those who had the greatest association with CCS progression during the follow-up period.

### Statistical analysis

Quantitative variables were described as mean and standard deviation or median and interquartile range, according to data normality. Categorical variables were described as absolute and relative frequencies.

To compare median change in the CCS, the Mann-Whitney or Kruskal-Wallis tests were applied, complemented by Dunn’s test. Normality was verified using the Kolmogorov-Smirnov test.

Associations with quantitative and ordinal variables were evaluated using Spearman’s correlation coefficient. Agreement between the 3 risk classifications was evaluated using the kappa coefficient.

Arterial age, FRS, and ASCVD were evaluated numerically using the ROC curve in relation to CCS greater than 400 at the second assessment.

The level of statistical significance considered was 5% (p < 0.05). The software used for data analysis was SPSS (Statistical Package for the Social Sciences), version 18.0.

## Results

The sample consisted of 150 men with a mean age of 58.2 years ( Table 1 ).

**Table 1:** – Characteristics of the study participants

Variables	First stage (n = 150)	Second stage (n = 139)
Age (years)	58.2 ± 5.3	65.7 ± 5.2
Years of schooling – n (%)		
≤ 8	86 (57.3)	79 (56.83)
9 to 12	39 (26.0)	37 (26.6)
> 12	25 (16.7)	23 (16.5)
Salary – n (%)		
< 5 times minimum wage	103 (68.7)	93 (66.9)
5 to 10 times minimum wage	37 (24.7)	40 (28.8)
> 10 times minimum wage	10 (6.7)	6 (4.3)
BMI (kg/m2)	26.8 ± 2.5	27.3 ± 3.4
Waist circumference (cm)	96.3 ± 7.9	97.7 ± 9.0
SBP (mmHg)	139.8 ± 12.2	137.36 ± 17.7
DBP (mmHg)	83.6 ± 7.8	78.1 ± 9.7
HR (bpm)	65.5 ± 10.7	66.4 ± 9.6
Tobacco use – n (%)	18 (12.0)	5 (3.6)
Family history of CAD – n (%)	23 (15.3)	40 (28.8)
Weekly physical activity – n (%)		
< 150 minutes	25 (16.7)	38 (27.3)
> 150 minutes	125 (83.3)	101 (72.7)
Altered physical examination – n (%)	14 (9.3)	17 (12.3)
Blood glucose (mg/dL)	106.0 ± 17.4	100.3 ± 19.5
Total cholesterol (mg/dL)	226.6 ± 38.0	195.7 ± 40.8
LDL cholesterol (mg/dL)	145.2 ± 32.8	120.6 ± 36.9
HDL cholesterol (mg/dL)	49.2 ± 13.2	50.2 ± 11.7
Triglycerides (mg/dL)	163.9 ± 141.2	124 ± 72.4
Coronary calcium score	94.4 ± 218.5 (0-83)	228.6 ± 468.5 (0-213)
No evidence	61 (40.7)	40 (28.7)
Mild (1 to 100)	57 (38.0)	52 (37.4)
Moderate (101 to 400)	21 (14.0)	25 (18.0)
High (401 to 1000)	10 (6.6)	14 (10.1)
Very high (> 1000)	1 (0.7)	8 (5.7)

* Quantitative data are described as mean ± standard deviation or median (25th to 75th percentiles) and categorical data are described as n (%). BMI: body mass index; CAD: coronary artery disease; DBP: diastolic blood pressure; HR: heart rate; SBP: systolic blood pressure.

During the study period, 11 follow-ups were lost: 6 of them died (no case of death from cardiovascular causes), and 5 declined to participate, leaving 139 individuals for the second stage.

Calculation of arterial age showed a difference of 1.7 years less in relation to chronological age, with an estimated mean arterial age of 56.5 years versus 58.2 years of chronological age. In the second stage, this difference increased to 3 years.

The restratification of cardiovascular risk using arterial age showed a statistically significant difference in relation to the stratification of cardiovascular risk by the FRS and ASCVD. We observed many individuals who were reclassified, as both higher and lower risk.

In relation to the FRS, 29% of individuals were reclassified to a higher risk category when using arterial age, while 34% remained in the same category, and 37% moved to a lower risk category. For the ASCVD, the findings were similar, with 33% remaining in the same category, while 31% moved to higher risk, and 36% moved to lower risk.

The greatest difference was observed in individuals with intermediate risk according to the FRS. Of the 107 individuals initially grouped in this category, only 27 (25.2%) remained in an intermediate risk category, while 52 (48.5%) were reclassified to low risk and 28 (26.2%) to high risk ( Figure 1 ).

**Figure 1:** – Reclassification of risk categories with different scores on the first evaluation.

When the risk categories were evaluated individually by the ASCVD classification, during the initial phase, there were 39 individuals classified as low risk, of whom 22 (56%) moved to a higher category. Of the 17 initially classified as high risk according to the ASCVD, only 5 remained (29%) as high risk after inclusion of the CCS.

During the second stage of the study, with an average time of 7.6 years after the first evaluation, a similar phenomenon occurred. In individuals classified as intermediate risk according to the FRS, the percentage who remained in the same classification was 30.5%, while 39.8% were reclassified to low risk and 29.6% to high risk ( Figure 2 ).

**Figure 2:** – Reclassification of risk categories with different scores on the second evaluation.

As an example, we cite the case of a 52-year-old individual with total cholesterol of 270 mg/dL, HDL-cholesterol of 43 mg/dL, systolic blood pressure of 129 mmHg, and diastolic blood pressure of 87 mmHg, with no history of smoking and without diabetes. When applying the FRS, the estimated risk was 8% (low risk), and ASCVD was 7.3 (borderline). However, the CCS was 195, which increased his risk according to arterial age to 30; therefore, he began to be considered as high risk for cardiovascular disease. In fact, during follow-up, his CCS increased to 631, a 323% increase, and only then was he considered high risk according to clinical criteria.

The agreement between the 3 scores (FRS, ASCVD, and arterial age) was also evaluated to classify risk, and it was observed that the kappa coefficient between arterial age and the FRS was 0.044 (p = 0.330); the kappa between arterial age and ASCVD was 0.073 (p = 0.080), and, between the FRS and ASCVD, it was 0.107 (p < 0.001), demonstrating significant agreement only between the FRS and ASCVD risk classifications, but with low, although significant agreement. In other words, the FRS and ASCVD classified risk groups in a similar manner, but both differed from the classification by arterial age.

There was a significant increase in the CCS in the period between the 2 assessments (first assessment: median =12.5; 25 to 75 percentiles: 0 to 81; second assessment: median = 33; 25 to 75 percentiles: 0 to 213; p < 0.001). The median variation in the CCS between the 2 assessments was 14 points (25 to 75 percentiles: 0 to 116).

When conducting progressive analysis of the change (delta) in the CCS, that is, when evaluating how much it varied over the follow-up period, we observed that individuals initially classified as low risk by arterial age had a very mild progression in the CCS, and this progression increased in proportion to higher risk estimated by the initial classification (p < 0.001). This relationship was not significant for the FRS (p = 0.862) or the ASCVD (p = 0.153). Differences between risk classifications with increasing CCS are displayed in Table 2 .

**Table 2:** – Differences between risk classifications according to change in calcium score

Variables	Total sample n (%)	∆ calcium score Median (P25-P75)	p
**Estimated risk classification by arterial age**			< 0.001
< 10 (low risk)	72 (48.0)	0 (0-11.8) ^a^	
10 to 20 (moderate risk)	39 (26.0)	38.5 (6-246) ^b^	
> 20 (high risk)	39 (26.0)	148 (58.5-466) ^c^	
**Framingham classification**			0.862
< 10% (low risk)	34 (22.7)	12 (0-76.5)	
10% to 20% (intermediate risk)	107 (71.3)	18.5 (0-126)	
> 20% (high risk)	9 (6.0)	47 (1-542)	
**ASCVD**			0.153
< 5% (low risk)	39 (26.0)	8.5 (0-35.0)	
5% to 19.9% (intermediate risk)	94 (62.7)	25 (0-173)	
≥ 20% (high risk)	17 (11.3)	30.6 (0.5-60.5)	

^a,b,c^ Equal letters do not differ by Dunn’s test at 5% significance. The numbers presented are relative to the score according to the Agatston method.

Figure 3 illustrates the differences found regarding the change in the calcium score according to the increase in cardiovascular risk by arterial age.

**Figure 3:** – Change in calcium score according to classification of estimated risk by arterial age. The line inside the box represents the median. The lower and upper limits of the box represent the 25th and 75th percentiles, respectively. The lower and upper error bars represent the estimated minimum and maximum values for the group. Circles and asterisks indicate the extreme values of the sample.

The numerical classification (% cardiovascular risk) estimated in the first evaluation by different methods (risk classification by arterial age, FRS, and ASCVD) was compared using the ROC curve for the outcome CCS > 400 in the second evaluation, which is considered a high degree of coronary calcification. That is, we aimed to evaluate the sensitivity and specificity of the methods as a way of detecting individuals who would develop a greater degree of coronary calcification during the follow-up period (7.6 years).

We observed that the classification by arterial age was significantly higher than the other methods, with an area under the curve of 0.870, while the ASCVD presented an area under the curve of 0.629, and the FRS was practically at the baseline, with 0.544 (Central Figure).

Individualized evaluation of the variables analyzed in the study showed that the vast majority had no statistically significant relationship with the change in the CCS. This relationship was not observed for age, presence of systemic arterial hypertension, statin use, diabetes mellitus, high LDL, hypertriglyceridemia, smoking or ex-smoking, physical activity, alcohol consumption, level of schooling, marital status, income, BMI, and waist circumference. Accordingly, we reduced the possibility of biases due to confounding factors.

There was a statistically significant positive relationship between the systolic blood pressure levels and the change in the CCS (p = 0.041); the higher the systolic blood pressure levels at baseline, the greater the increase in the CCS ( Table 3 ).

**Table 3:** – Associations of variables with change in calcium score

Quantitative variable	∆ calcium score Spearman’s correlation coefficient	p
Systolic blood pressure	0.174	0.041
**Qualitative variable**	**Median (P25-P75)**	**p**
Low HDL		0.041
No	13 (0-114)	
Yes	457.5 (368-547)	

SBP: systolic blood pressure.

Individuals with low HDL showed a significantly greater increase in the calcium score, as shown in Table 3 . Their average increase in the CCS during the study was 457, whereas, in the remaining individuals with normal HDL, the average increase in the calcium score was 13.

## Discussion

The CCS is an indicator of subclinical atherosclerosis, and elevated values indicate greater degree of atherosclerosis. ^9 , 11 , 12^

The impact of traditional risk factors is not uniform among individuals, which is why traditional scores do not always have sufficient sensitivity and specificity for correct risk stratification. ^4 - 6^

Studies have shown that incorporating arterial age into traditional risk scores increases the area under the curve, which improves the sensitivity and specificity of this score in determining cardiovascular risk. ^11 , 25^

Our study has shown that a significant portion of individuals are restratified into different risk categories when evaluated using arterial age. The percentage whose risk category changed was greater than that observed in the PAAC study ^26^ and the Heinz Nixdorf Recall Study, ^27^ an assessment derived from the MESA study. The category that underwent the most changes is the one classified as intermediate by traditional risk scores, which is similar to the studies mentioned above, where between 1 in 4 and 1 in 6 individuals moved to a higher risk category. In our study, we observed a greater number of individuals who moved to a lower risk category in comparison to the cited studies.

These findings reinforce the indication that already exists in the guidelines for CCS to be determined, especially in individuals with intermediate cardiovascular risk according to the traditional risk scores. ^22 , 28 , 29^

Furthermore, the most objective piece of data was that the classification of cardiovascular risk by arterial age showed a direct relationship with increased coronary calcification determined using CCS during follow-up. Individuals initially classified as low risk by arterial age had a very mild CCS progression, and this progression increased in proportion to higher risk estimated by the initial classification. This relationship had already been described in the literature for major cardiovascular outcomes; nonetheless, there is no description of the proportional increase in the CCS based on the initial classification by arterial age. These are the most relevant data from our study, and they may indicate performance of CCS, even in individuals with low risk according to traditional scores, keeping in mind that, even among these individuals, some had a higher arterial age and more significant CCS progression throughout the follow-up.

Among all the risk factors studied, high systolic blood pressure and low HDL were the ones that were significantly associated with the greatest CCS progression throughout the study.

HDL cholesterol has already shown to be an important cardiovascular risk factor when at low levels, regardless of other cholesterol levels. ^30 , 31^ In our study, it was the isolated factor with the greatest impact on the progression of coronary calcification.

Although a significant reclassification of cardiovascular risk occurred, it was not possible to evaluate the potential reduction in cardiovascular outcomes in this study population. This fact makes us question the applicability of the method to the general population, bearing in mind that the costs and potential risks associated with the test cannot be ignored.

The main study limitations are the relatively low number of participants, the absence of women in the sample, and the inability to show major cardiovascular outcomes (acute myocardial infarction, death of cardiovascular origin, or stroke). Furthermore, participants were volunteers and were selected by convenience.

One of the potential biases to be considered is the use of statins and their possible effect on coronary calcium. It has been established that the use of statins can increase the CCS; therefore, the progression of the CCS is not always something undesirable, given that it can only translate “stabilization” of the existing coronary plaques. We had information on the patients who used statins in both stages of the study, although we did not obtain information about the time and continuity of use.

Furthermore, the equipment used between the 2 study stages was different, which could cause a potential measurement bias.

An important limitation to be considered is the absence of women in the study. We are aware of the importance of including both sexes for more reliable analysis and so that the data can be generalized. Future research should be conducted including women to provide a more comprehensive understanding of the factors that influence vascular age and cardiovascular risk classifications.

Notwithstanding the potential biases, we consider that the factor of CCS progression was quite significant, and it had a direct relationship with the initial classification based on arterial age. Furthermore, elevated systolic blood pressure and low levels of HDL-cholesterol were associated with greater CCS progression.

## Conclusion

Cardiovascular risk stratification using arterial age was more capable of identifying individuals with elevated risk of atherosclerosis progression measured by the CCS than traditional risk scores (FRS and ASCVD). The individual variables most associated with CCS progression were high systolic blood pressure and low HDL.
